# Race, socioeconomic deprivation, and hospitalization for COVID-19 in English participants of a national biobank

**DOI:** 10.1186/s12939-020-01227-y

**Published:** 2020-07-06

**Authors:** Aniruddh P. Patel, Manish D. Paranjpe, Nina P. Kathiresan, Manuel A. Rivas, Amit V. Khera

**Affiliations:** 1 Center for Genomic Medicine and Division of Cardiology, Department of Medicine, Massachusetts General Hospital, Boston, MA USA; 2 Harvard Medical School, Boston, MA USA; 3 Cardiovascular Disease Initiative, Broad Institute of MIT and Harvard, Cambridge, MA USA; 4 Milton Academy, Milton, MA USA; 5 Department of Biomedical Data Science, Stanford University, Stanford, CA USA

**Keywords:** Race, Health disparity, Socioeconomic deprivation, COVID-19

## Abstract

Preliminary reports suggest that the Coronavirus Disease 2019 (COVID− 19) pandemic has led to disproportionate morbidity and mortality among historically disadvantaged populations. We investigate the racial and socioeconomic associations of COVID− 19 hospitalization among 418,794 participants of the UK Biobank, of whom 549 (0.13%) had been hospitalized. Both Black participants (odds ratio 3.7; 95%CI 2.5–5.3) and Asian participants (odds ratio 2.2; 95%CI 1.5–3.2) were at substantially increased risk as compared to White participants. We further observed a striking gradient in COVID− 19 hospitalization rates according to the Townsend Deprivation Index − a composite measure of socioeconomic deprivation − and household income. Adjusting for socioeconomic factors and cardiorespiratory comorbidities led to only modest attenuation of the increased risk in Black participants, adjusted odds ratio 2.4 (95%CI 1.5–3.7). These observations confirm and extend earlier preliminary and lay press reports of higher morbidity in non-White individuals in the context of a large population of participants in a national biobank. The extent to which this increased risk relates to variation in pre-existing comorbidities, differences in testing or hospitalization patterns, or additional disparities in social determinants of health warrants further study.

## Background

The Coronavirus Disease 2019 (COVID-19) pandemic has led to substantial morbidity and mortality globally, but initial reports suggest historically disadvantaged populations may be most afflicted. For example, a preliminary report from the United States Centers for Disease Control and Prevention indicated that 33% of hospitalized patients were Black as compared to 18% of the U.S. population [1]. Within the densely populated and largely Hispanic suburb of Chelsea, Massachusetts, serologic tests suggested that up to 32% of individuals had been exposed [2].

The extent to which these disparities are related to socioeconomic versus biologic factors is largely unknown.

## Methods

The UK Biobank is a prospective cohort study that recruited over 500,000 middle-aged individuals between the years 2006 and 2010, allowing for linkage of extensive baseline, genetic and clinical data [3]. Recently, COVID-19 testing results for a subset of participants in England were made available [4]. Individuals were excluded from this analysis if they had enrolled outside England, died prior to 2019, or had an outpatient test positive for COVID-19, in whom the clinical trajectory was uncertain. Additional details are provided in the Additional file 1.

To quantify the relationship between race and hospitalization for COVID-19, 418,794 UK Biobank participants were studied. Mean age at last date of follow-up was 65.8 years, and 188,914 (45%) were male. 400,438 (95.6%) self-reported as White, 10,642 (2.5%) as Asian, and 7412 (1.8%) as Black. 549 (0.13%) individuals were hospitalized with a positive test for COVID-19, detected at 17 assessment centers across England.

## Results

**Fig. 1:**
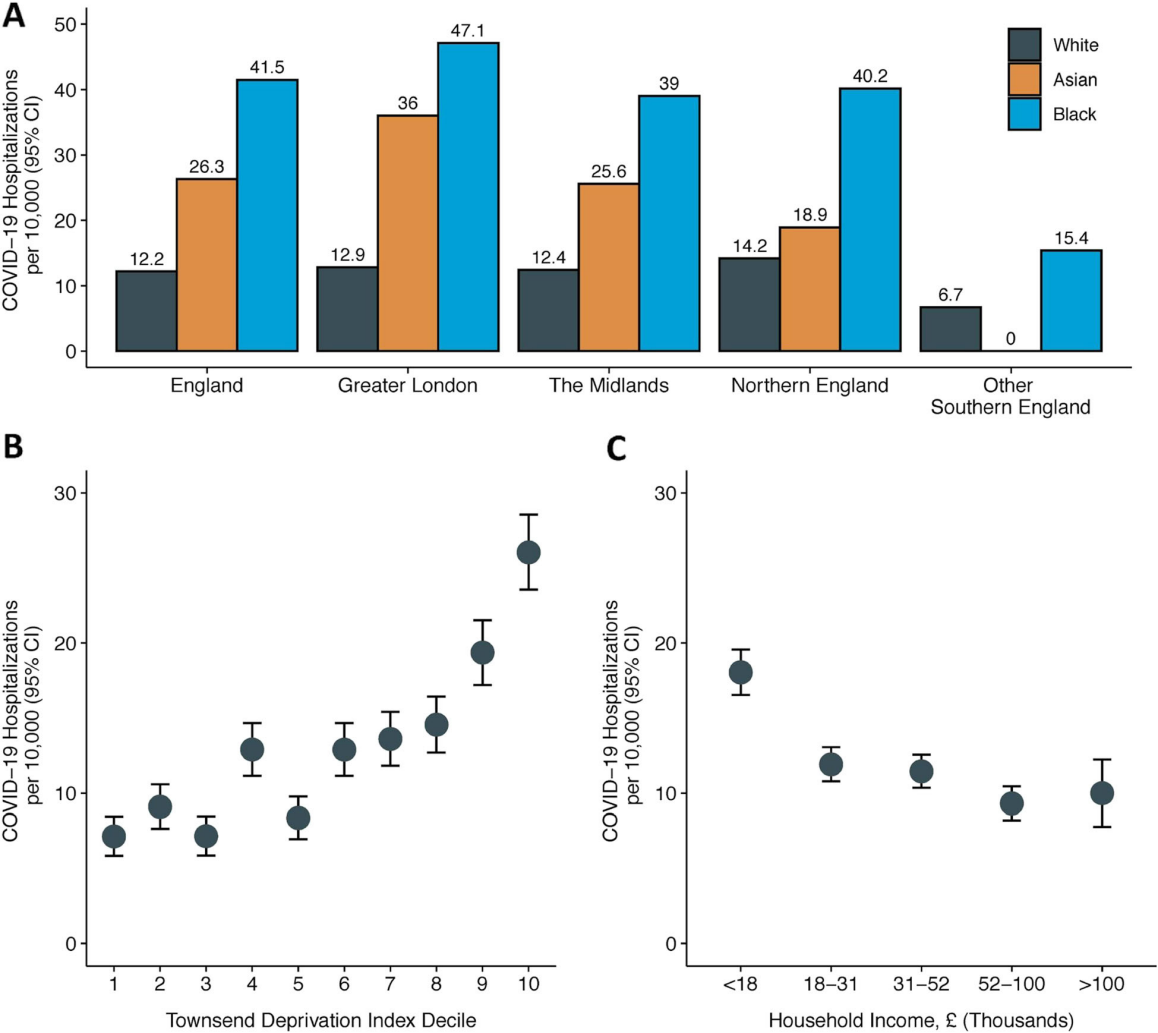
COVID-19 hospitalization rates by race and geographic region, Townsend Deprivation Index, and household income. **a** COVID-19 hospitalizations per 10,000 individuals stratified by self-reported race and geographic region grouped based on socio-demographic similarities. In all regions, significant differences in COVID-19 hospitalizations were observed between self-reported White, Asian and Black individuals (1-way ANOVA *p* < 0.001). **b** Mean COVID-19 hospitalizations per 10,000 individuals by deciles of the Townsend Deprivation Index. Townsend Deprivation Index was significantly associated with COVID-19 hospitalization in adjusted and unadjusted models (*p* < 0.001). **c** COVID-19 hospitalizations per 10,000 individuals by brackets of self-reported pre-tax household income. Pre-tax household income was significantly associated with COVID-19 hospitalization in an unadjusted model (*p* < 0.001)

COVID-19 hospitalization was noted in 32 of 7714 (0.4%) Black participants, 28 of 10,614 (0.2%) Asian participants, and 489 of 400,438 (0.1%) White participants, with results largely consistent across English regions (Fig. 1a). In a logistic regression model adjusted for age, sex, and geographic region, both Black participants (odds ratio 3.7; 95%CI 2.5–5.3) and Asian participants (odds ratio 2.2; 95%CI 1.5–3.2) were at increased risk as compared to White participants.

To understand the relationship between socioeconomic status and COVID-19 hospitalization, individuals were stratified at enrollment into deciles of the Townsend Deprivation Index, a previously validated composite metric based on employment status, car or home ownership, and household crowding [5]. Participants with greater Townsend Deprivation Indices were at substantially higher risk of COVID-19 hospitalization, with a similar pattern observed based on self-reported household income (Fig. 1b, c).

The relationship between race and COVID-19 hospitalization was modestly attenuated in a logistic regression model that additionally adjusted for Townsend Deprivation Index, household income, hypertension, diabetes, heart failure, ischemic stroke, dementia, chronic obstructive pulmonary disease, prior pneumonia, dementia, chronic kidney disease, smoking status, statin use, and body mass index--odds ratios for Black and Asian participants of 2.4 (95%CI 1.5–3.7) and 1.8 (95%CI 1.1–2.9) as compared to White participants respectively. Unadjusted and adjusted models are available in the Additional file 1.

## Discussion

Within a large population of participants in a national biobank, striking gradients in risk of hospitalization for COVID-19 were noted according to race and a metric of socioeconomic deprivation. The increased risk observed in Black participants was attenuated but remained significant after adjusting for socioeconomic deprivation, household income, as well as cardiometabolic and respiratory comorbidities. The extent to which this increased risk relates to variation in pre-existing comorbidities, differences in testing or hospitalization patterns, or additional disparities in social determinants of health warrants further study [6]. With respect to potential biologic factors, ongoing efforts seek to determine whether genetics--known to both vary substantially across racial groups and contribute to pre-existing comorbidities--plays an important role in COVID-19 disease severity.

This study has limitations. First, the UK Biobank enrolled individuals on a volunteer basis and is not a population-based study--additional efforts are needed to generalize these observations in other settings. Second, Townsend Deprivation Index and household income were assessed at enrollment, and participants’ status may have changed in subsequent years. Third, disease endpoints were ascertained through participant self-reporting, diagnosis codes from hospitalizations, and national death registries. Fourth, additional and more sophisticated analytic techniques may prove useful in further disentangling COVID-19 related disparities.

## Supplementary information

**Additional file 1.**

## Data Availability

Data is made available from the UK Biobank to researchers from universities and other research institutions with genuine research inquiries following IRB and UK Biobank approval.
