# Supplementary material for: Race, socioeconomic deprivation, and hospitalization for COVID-19 in English participants of a national biobank
Source: Int J Equity Health. 2020 Jul 6;19:114. doi: 10.1186/s12939-020-01227-y (PMC7336098; doi:10.1186/s12939-020-01227-y)
Supplement: Supplementary file 1 — Additional file 1. [file 12939_2020_1227_MOESM1_ESM.docx]

**APPENDIX**

**Supplementary Methods:**

*Study populations*

The UK Biobank is an observational study of over 500,000 middle-aged individuals in the United Kingdom enrolled between the ages of 40 and 69 years between 2006 and 2010 with regular interval follow up.(1,2) Self-reported data from a detailed questionnaire on enrollment was used to calculate the Townsend Deprivation Index, which is a composite metric of unemployment, lack of car or home ownership, and household overcrowding by geographical area.(3) Racial groups were defined as self-reported white, Asian, or black. White included white-British, Irish, and any other white background. Asian included individuals of Asian-British, Indian, Pakistani, Bangladeshi, Chinese, and other Asian backgrounds. Black included black-British, black-Caribbean, African, and other black populations. History of hypertension, diabetes, coronary artery disease, heart failure, ischemic stroke, chronic obstructive pulmonary disease, prior pneumonia, Alzheimer’s disease or dementia, and chronic kidney disease was determined based on ICD-9 and ICD-10 codes from self-report, hospitalization records, and death registry. Body mass index was measured at the enrollment visit. Smoking history, alcohol consumption, and statin use were determined from initial detailed questionnaire.

20,442 individuals that had died prior to September 2019 were excluded. Thereafter, 97 individuals with positive tests who did not have any indicators of hospitalization were excluded as their illness trajectory remains unknown. 53,983 individuals in the Biobank who were assessed at centers outside of England were removed as COVID-19 testing data collection at these sites has been limited. An additional 9,240 individuals who reported mixed race or did not report a race were excluded, resulting in a final study population of 418,794 individuals. 549 individuals were found to have a positive test and an associated hospitalization. Based on testing and hospitalization patterns, this cohort was deemed to have severe symptoms requiring hospitalization and was assumed to be comprehensive given dynamic linking of hospital and testing data with the UK Biobank database, as below.(4) In subsequent analysis, the 549 individuals with COVID-19 hospitalization were compared to the remaining 418,245 individuals. The 418,245 individuals without documented positive tests and hospitalization status were assumed to be a collection of uninfected individuals, asymptomatic virus carriers, and individuals with mildly symptomatic COVID-19 infection.

Informed consent was obtained from all participants. Analysis of UK Biobank data was approved by the Partners Healthcare institutional review board (application number 7089).

*Regions of United Kingdom*

Geographic regions were obtained by mapping the UK Biobank assessment center to one of 9 Nomenclature of Territorial Units for Statistics first-level regions of England: North East, North West, Yorkshire and the Humber, East Midlands, West Midlands, London, South East and South West). No data was available for East of England. Regions were combined based on socioeconomic and geographic characteristics using the following scheme:

North East, North West, Yorkshire and the Humber: Northern England

East Midlands and West Midlands: The Midlands

London: Greater London

South West, South East: Other South

*COVID-19 Testing*

Nose swabs, throat swabs, and lower respiratory sputum samples were collected from individuals across centers in England between March 16, 2020 and April 14, 2020. Testing in this period was largely restricted to those with symptoms in hospital. A polymerase chain reaction assay was performed on collected samples to detect the presence of severe acute respiratory syndrome coronavirus 2, and results were dynamically linked to the Biobank database database.[^4^](https://www.zotero.org/google-docs/?hOW7BZ) Several individuals had multiple tests, and presence of any single positive test associated with hospitalization determined them to be a case.

*Statistical Analysis*

For each variable of interest (race, Townsend Deprivation Index, and income) an unadjusted model for the association between the variable and COVID19 hospitalization was fit using binomial logistic regression (Model 1). A fully adjusted binomial logistic regression model was fit by including covariates of age, sex, race, geographical region, Townsend Deprivation Index, income, cardiometabolic and respiratory comorbidities (Model 2). P values < 0.05 were deemed as significant and significance after accounting for multiple hypothesis testing (Benjamini-Hochberg *P* < 0.05) was determined. Statistical analyses were performed with the use of R software, version 3.5 (R Project for Statistical Computing).

**Statistical Model 1:** Basic regression analyses with age, sex, race, and geographic region

|  | **Unadjusted Model** | | **Adjusted Model** | |
| --- | --- | --- | --- | --- |
|  | OR (95% CI) | P Value | OR (95% CI) | P Value |
| (Intercept) | 0 (0-0) | <0.001 | 0 (0-0) | <0.001* |
| Age | 1.02 (1.01-1.03) | <0.001 | 1.02 (1.01-1.04) | <0.001* |
| Sex | 1.53 (1.3-1.81) | <0.001 | 1.52 (1.28-1.8) | <0.001* |
| Race |  |  |  |  |
| White (Reference) | 1 |  | 1 |  |
| Asian | 2.16 (1.47-3.16) | <0.001 | 2.16 (1.46-3.2) | <0.001* |
| Black | 3.41 (2.38-4.88) | <0.001 | 3.65 (2.51-5.31) | <0.001* |
| Region |  |  |  |  |
| Greater London (Reference) | 1 |  | 1 |  |
| Northern England | 0.86 (0.68-1.07) | 0.17 | 0.99 (0.79-1.26) | 0.95 |
| Other Southern England | 0.40 (0.29-0.55) | <0.001 | 0.47 (0.33-0.65) | <0.001* |
| The Midlands | 0.79 (0.6-1.04) | 0.09 | 0.88 (0.66-1.16) | 0.36 |

*P value significant after accounting for multiple hypothesis testing (Benjamini-Hochberg *P* < 0.05)

**Statistical Model 2:** Regression analyses with cardiometabolic and respiratory comorbidities

|  | **Unadjusted Model** | | **Adjusted Model** | |
| --- | --- | --- | --- | --- |
| **Feature** | OR (95% CI) | P Value | OR (95% CI) | Adjusted  P Value |
| (Intercept) | 0 (0-0) | <0.001 | 0 (0-0) | <0.001* |
| Age | 1.02 (1.01-1.03) | <0.001 | 1.02 (1-1.03) | 0.02 |
| Sex | 1.53 (1.3-1.81) | <0.001 | 1.37 (1.12-1.66) | 0.002* |
| Race |  |  |  |  |
| White (Reference) | 1 |  | 1 |  |
| Asian | 2.16 (1.47-3.16) | <0.001 | 1.75 (1.08-2.85) | 0.02 |
| Black | 3.41 (2.38-4.88) | <0.001 | 2.38 (1.52-3.74) | <0.001* |
| Region |  |  |  |  |
| Greater London (Reference) | 1 |  | 1 |  |
| Northern England | 0.86 (0.68-1.07) | 0.16926 | 1.14 (0.86-1.5) | 0.37 |
| Other Southern England | 0.40 (0.29-0.55) | <0.001 | 0.67 (0.46-0.98) | 0.04 |
| The Midlands | 0.79 (0.6-1.04) | 0.12 | 1.14 (0.82-1.58) | 0.43 |
| Coronary Artery Disease | 1.83 (1.4-2.39) | <0.001 | 0.95 (0.67-1.36) | 0.79 |
| Hypertension | 1.72 (1.45-2.03) | <0.001 | 1.12 (0.9-1.39) | 0.32 |
| Diabetes Mellitus | 2.66 (2.13-3.32) | <0.001 | 1.52 (1.14-2.03) | 0.005* |
| Heart Failure | 2.24 (1.29-3.89) | 0.004 | 1.09 (0.56-2.14) | 0.79 |
| Ischemic Stroke | 1.42 (0.64-3.18) | 0.39 | 0.94 (0.39-2.3) | 0.90 |
| Body Mass Index | 1.07 (1.05-1.08) | <0.001 | 1.04 (1.03-1.06) | <0.001* |
| Chronic Obstructive Pulmonary Disease | 2.67 (1.94-3.67) | <0.001 | 1.51 (1-2.28) | 0.05 |
| Previous Pneumonia | 1.81 (1.24-2.65) | 0.002 | 1.31 (0.83-2.05) | 0.25 |
| Alzheimer’s Disease or Dementia | 4.24 (0.59-30.31) | 0.15 | 5.08 (0.7-36.68) | 0.11 |
| Chronic Kidney Disease | 3.55 (2.29-5.49) | <0.001 | 2.01 (1.19-3.41) | 0.01 |
| Current Smoker | 1.04 (0.79-1.37) | 0.77 | 0.91 (0.66-1.25) | 0.55 |
| Statin Usage | 1.88 (1.55-2.27) | <0.001 | 1.06 (0.82-1.38) | 0.64 |
| Alcohol Consumption | 1.13 (1.07-1.19) | <0.001 | 1.04 (0.98-1.11) | 0.21 |
| Townsend Index | 1.13 (1.1-1.16) | <0.001 | 1.09 (1.05-1.12) | <0.001* |
| Average Income | 0.83 (0.77-0.9) | <0.001 | 1.01 (0.92-1.11) | 0.76 |

*P value significant after accounting for multiple hypothesis testing (Benjamini-Hochberg *P* < 0.05)

**REFERENCES:**

1. Bycroft C, Freeman C, Petkova D, Band G, Elliott LT, Sharp K, et al. The UK Biobank resource with deep phenotyping and genomic data. Nature. 2018;562(7726):203–9.

2. Sudlow C, Gallacher J, Allen N, Beral V, Burton P, Danesh J, et al. UK biobank: an open access resource for identifying the causes of a wide range of complex diseases of middle and old age. PLoS Med. 2015 Mar;12(3):e1001779.

3. Townsend P, Phillimore P, Beattie A. Health and Deprivation: Inequality and the North. London, England: Croom Helm; 1988.

4. Armstrong J, Rudkin J, Allen N, Crook D, Wilson D, Wyllie D, et al. Dynamic linkage of COVID-19 test results between Public Health England’s Second Generation Surveillance System and UK Biobank. Microbial Genomics. 2020;
